# Waist circumference and insulin resistance: a cross-sectional study of Japanese men

**DOI:** 10.1186/1472-6823-9-1

**Published:** 2009-01-12

**Authors:** Shinji Tabata, Shinichiro Yoshimitsu, Tadamichi Hamachi, Hiroshi Abe, Keizo Ohnaka, Suminori Kono

**Affiliations:** 1 Self-Defense Force Fukuoka Hospital, Fukuoka, Japan; 2 Department of Preventive Medicine, Faculty of Medical Sciences, Kyushu University, Fukuoka, Japan; 3 Department of Geriatric Medicine, Faculty of Medical Sciences, Kyushu University, Fukuoka, Japan

## Abstract

**Background:**

Visceral obesity is positively related to insulin resistance. The nature of the relationship between waist circumference and insulin resistance has not been known in Japanese populations. This study examined the relationship between waist circumference and insulin resistance and evaluated the optimal cutoff point for waist circumference in relation to insulin resistance in middle-aged Japanese men.

**Methods:**

Study subjects included 4800 Japanese men aged 39 to 60 years. Insulin resistance was evaluated by the homeostasis model assessment of insulin resistance (HOMA-IR). The relationship of waist circumference with HOMA-IR was assessed by use of adjusted means of HOMA-IR and odds ratios of elevated HOMA-IR defined as the highest quintile (≥2.00). Receiver operating characteristics (ROC) curve analysis using Youden index and the area under curve (AUC) was employed to determine optimal cutoffs of waist circumference in relation to HOMA-IR.

**Results:**

Adjusted geometric means of HOMA-IR and prevalence odds of elevated HOMA-IR were progressively higher with increasing levels of waist circumference. In the ROC curve analysis, the highest value of Youden index was obtained for a cutoff point of 85 cm in waist circumference across different values of HOMA-IR. Multiple logistic regression analysis also indicated that the AUC was consistently the largest for a waist circumference of 85 cm.

**Conclusion:**

Waist circumference is linearly related to insulin resistance, and 85 cm in waist circumference is an optimal cutoff in predicting insulin resistance in middle-aged Japanese men.

## Background

Visceral obesity is closely linked to insulin resistance, and is currently regarded as a principle component of the metabolic syndrome [1,2]. It is well documented that insulin resistance is predictive of the risk of type 2 diabetes and cardiovascular disease [3-6]. In conjunction with worldwide recognition of the metabolic syndrome [2], the size of waist circumference as an estimate of visceral obesity has been a matter of controversy. The International Diabetes Federation (IDF) has adopted different cutoffs for waist circumference in different ethnicities [7]; the cutoff points for Europids are 94 cm in men and 80 cm in women while those for Chinese and South Asians are 90 in men and 80 in women. The cutoff points for Japanese are set at 85 cm in men and 90 cm in women [7]. These cutoffs for Japanese were derived from the criteria for the metabolic syndrome proposed by an expert group of obesity research in Japan [8]. This recommendation was based on the findings from a cross-sectional study of 1200 men and women that waist circumferences corresponding to 100 cm^2 ^of visceral fat area were 84.4 cm in men and 92.5 cm in women [8]. The visceral fat area of 100 cm^2 ^obtained from computed tomography was postulated as a threshold of the accumulation of obesity-related diseases [8]. Several cross-sectional studies have evaluated appropriateness of the cutoffs proposed for Japanese, showing optimal cutoffs of 85–87 cm for men and 80–82 cm for women [9-11]. However, these studies [9-11] as well as the original study [8] were primarily based on the relationship between waist circumference and multiple components other than abdominal obesity of the metabolic syndrome. These definitions have not been directly examined in relation to type 2 diabetes or insulin resistance.

Furthermore, the nature of the relationship between waist circumference and insulin resistance or hyperinsulinemia has not been examined in Japanese populations. We examined the relationship between waist circumference and insulin resistance in a large population of middle-aged Japanese men. In this study, we aim to clarify whether there is a threshold in waist circumference in relation to insulin resistance and whether the proposed cutoff of waist circumference for Japanese men is appropriate.

## Methods

### Study subjects

Study subjects were male officials in the Self-Defense Forces (SDF) who received a pre-retirement health examination from January 1997 to March 2002, health check-up at age 50 years from April 2002 to September 2006, or health check-up at age 40 years from April 2005 to September 2006 at the Self-Defense Force Fukuoka Hospital. The pre-retirement health examination was a nationwide program offering a comprehensive medical examination to those retiring from the SDF. The health check-ups at age 40 and 50 years are also a nationwide program, including almost the same items of examinations as done in the pre-retirement health examination. The health check-up at age 50 years was substituted for the pre-retirement health examination in April 2002, and the health check-up at age 40 years was newly introduced in April 2005. These health examinations included abdominal ultrasonography, 75-g oral glucose tolerance test and blood biochemistry among others, as described in detail elsewhere [12,13]. The study was approved by the ethics committee of Kyushu University Faculty of Medical Sciences.

In a consecutive series of 5423 men during the above-mentioned period, 301 men refused to participate in the survey. Excluded were those with morbid conditions affecting glucose metabolism or insulin levels. Thus, of the remaining 5122 men, 200 were excluded because of a prior history of cancer (*n *= 61), newly diagnosed cancer (*n *= 12), prevalent conditions such as thyroid disease (*n *= 20), chronic hepatitis or liver cirrhosis (*n *= 71), and chronic kidney disease (*n *= 14), use of steroids (*n *= 17) or insulin (*n *= 20); some men had more than one condition for exclusion. We also excluded 122 men in whom fasting plasma insulin or glucose was not determined (*n *= 121) and waist circumference was not measured (*n *= 1). A total of 4800 men remained in the analysis. We did not exclude individuals with oral medication for diabetes mellitus so as to maximize the number of subjects with insulin resistance in the analysis. Insulin resistance status is probably not affected measurably by oral medication for diabetes mellitus [14].

### Procedures

Venous blood was sampled after an overnight fast for biochemical measurements. Plasma glucose and insulin were determined by the glucose oxidase method and the enzyme immunoassay, respectively, using commercial kits at the hospital laboratory. Assay kits were obtained from different sources during the period, but standardization was done routinely on the introduction of new assay kits. Insulin resistance was evaluated by the homeostasis model assessment of insulin resistance (HOMA-IR) [15]. This measure reportedly explains 65% of insulin sensitivity measured by the glucose clamp technique [16]. Waist circumference was measured in the horizontal plane at the umbilical level by nurses with a subject wearing underwear in a standing position.

A self-administered questionnaire was used to ascertain alcohol use, smoking habits, and other lifestyle characteristics. Details of the questionnaire have been described previously [12,13]. In brief, smokers were defined as those who had ever smoked cigarettes daily for at least one year. Former smokers were separated from lifelong nonsmokers, and current and past smokers reported the average number of cigarettes smoked per day. Cigarette smoking was classified into five categories (never, past, and current with a consumption of < 20, 20–24, or ≥ 25 cigarettes per day). Alcohol drinkers were defined as those having drunk alcoholic beverages at least once per week for one year or longer, and former alcohol use was separated from lifetime non-use of alcohol. Daily ethanol intake was estimated for current drinkers on the basis of consumption frequencies and amounts of five types of alcoholic beverages (sake, shochu, beer, whisky/brandy and wine) on average in the past year. Alcohol use was categorized into never, past, and current with a consumption of < 30, 30–59, or ≥ 60 ml of ethanol per day. Medical history and current medication were ascertained by ward nurses and physicians.

### Statistical analysis

The relationship of waist circumference with HOMA-IR was statistically assessed by use of adjusted means of HOMA-IR and odds ratios of elevated HOMA-IR arbitrarily defined as the highest quintile (≥2.00). The adjusted means were calculated by analysis of covariance, and the odds ratios with 95% confidence intervals were obtained by the logistic regression analysis. The distribution of HOMA-IR was skewed to the right side, and the values were transformed to the natural logarithms in the analysis. Thus the presented means were always geometric means. Statistical adjustment was made for age, rank in the SDF (three classes), smoking, and alcohol use. Trend of the association was assessed with ordinal scores assigned to the levels of waist circumference.

Receiver operating characteristics (ROC) curve analysis was employed to determine optimal cutoffs of waist circumference in relation to insulin resistance defined by different values of HOMA-IR, with and without allowance for the covariates. In the analysis without consideration to the covariates, the optimal cutoff point was obtained by the Youden index, *i.e.*, maximum (sensitivity + specificity -1) [17]. After logistic regression analysis controlling for the covariates, ROC curve was depicted and area under ROC curve (AUC) was calculated for each of the cutoffs of 80, 85, and 90 cm in waist circumference. Statistical significance was declared if a two-sided *P *value was less than 0.05 or if the 95% confidence intervals did not include unity. All computations were mostly performed using the SAS version 8.2 (SAS Institute Inc., Cary, NC). The ROC curve analysis was done by using Stata SE version 8 (Stata, College Station, TX).

## Results

**Figure 1:**
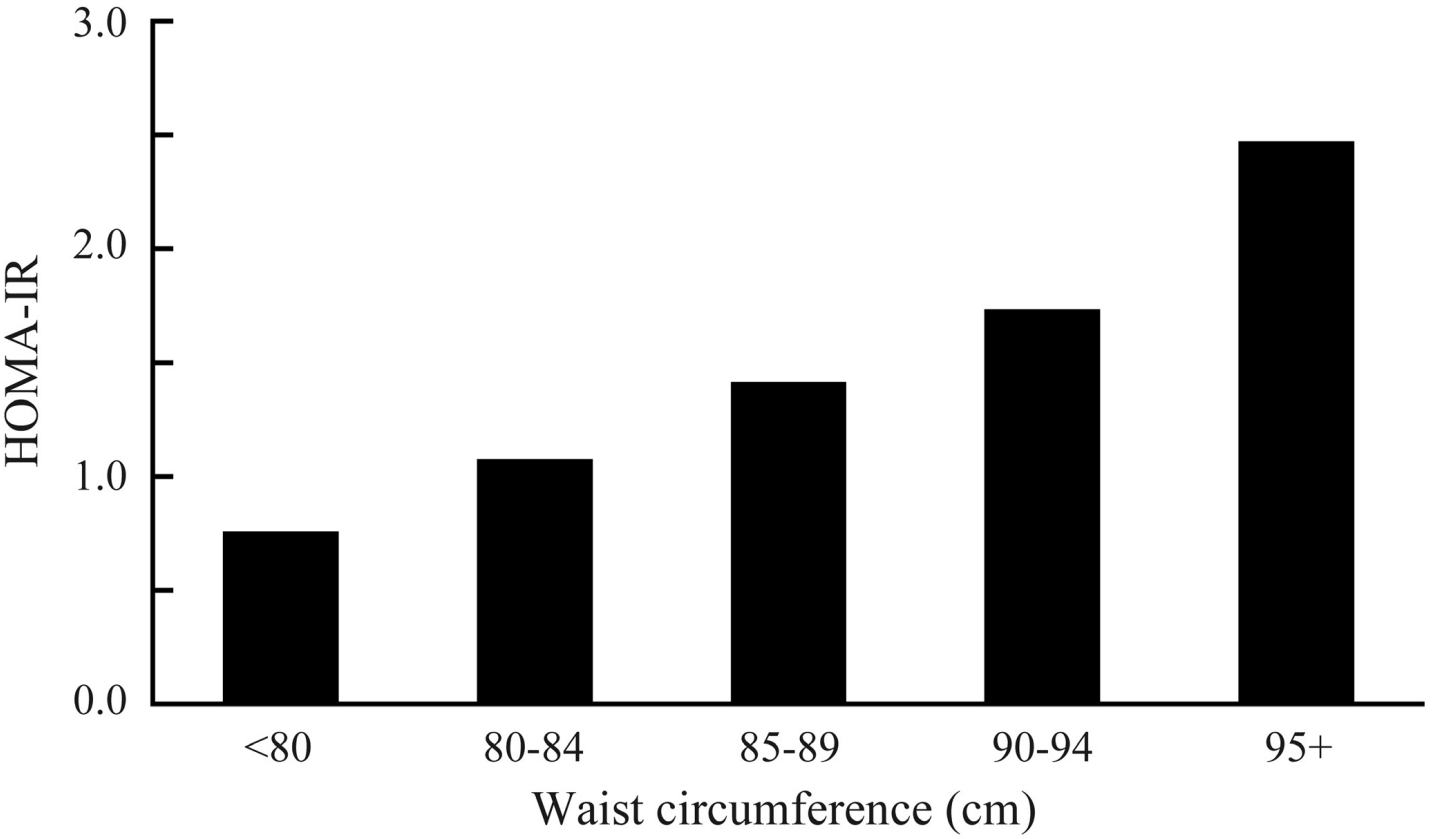
**Age-adjusted geometric means of HOMA-IR according to categories of waist circumference**. HOMA-IR, homeostasis model assessment of insulin resistance.

Age ranged 39 to 60 years, with a mean of 51. Characteristics of the study subjects are described in Table 1. Spearman correlation coefficient between waist circumference and HOMA-IR was 0.52. As shown in Figure 1, geometric means of HOMA-IR were progressively higher with increasing levels of waist circumference; age-adjusted means for the waist circumference of < 80, 80–84, 85–89, 90–94, and ≥ 95 were 0.76, 1.07, 1.42, 1.73, and 2.49 (trend *P *< 0.0001). Adjustment for rank in the SDF, smoking, and alcohol use did not change the relation; adjusted geometric means from the lowest to highest categories of waist circumference were 0.76, 1.08, 1.42, 1.74, and 2.46, respectively (trend *P *< 0.0001).

**Table 1:** Characteristics of the study subjects

Variable	Value
Age (year), mean (SD)	50.5 (3.7)
Body mass index (kg/m^2^), mean (SD)	24.0 (2.7)
Waist circumference (cm), mean (SD)	83.7 (7.2)
Current smoking (%)	48.2
Alcohol use (%)	66.1
Physical activity (MET-hours/week)*, median (IQR)	16 (5–27)
Serum total cholesterol (mg/dL), mean (SD)	206.7 (34.5)
Serum HDL cholesterol (mg/dL), mean (SD)	57.2 (16.4)
Serum triglycerides (mg/dL), median (IQR)	127 (91–178)
Fasting plasma glucose (mmol/L), median (IQR)	5.6 (5.2–6.0)
Fasting plasma insulin (pmol/L), median (IQR)	34 (22–50)
HOMA-IR, median (IQR)	1.18 (0.77–1.80)
History of myocardial infarction (%)	0.5
History of stroke (%)	0.9
Use of lipid-lowering drug (%)	3.1
Use of antihypertensive drug (%)	9.9
Oral medication for diabetes mellitus (%)	1.3

* Leisure-time physical activity.

IQR, interquartile range; HOMA-IR, homeostasis model assessment of insulin resistance; MET, metabolic equivalent.

The prevalence odds of elevated HOMA-IR (≥ 2.00) also increased stepwise with increasing circumferences of the waist (Table 2). Men with a waist of 80–84 cm showed a statistically significant increase in the odds ratio as compared with the lowest category of waist circumference (< 80 cm). Waist circumference of 90–94 was associated with a 15-fold increase in the odds ratio of elevated HOMA-IR, and a 45-fold increase in the odds ratio was noted for the highest category (≥ 95 cm).

**Table 2:** Age-adjusted and multivariate-adjusted odds ratios of elevated HOMA-IR according to categories of waist circumference

	Number of men (%)		Odds ratio (95% confidence interval)	
		
Waist (cm)	Total	Elevated HOMA-IR*	Age-adjusted	Multivariate-adjusted†
< 80	1375	58 (4.2)	1.0 (referent)	1.0 (referent)
80–84	1307	156 (11.9)	3.0 (2.2–4.2)	3.2 (2.3–4.3)
85–89	1141	296 (25.9)	7.8 (5.8–10.5)	8.2 (6.1–11.0)
90–94	645	247 (38.3)	13.9 (10.3–19.0)	15.2 (11.1–20.8)
95+	332	216 (65.1)	42.2 (29.8–59.7)	45.2 (31.8–64.4)

* The top quintile of HOMA-IR (≥ 2.00).

† Adjusted for age, rank in the Self-Defense Forces, smoking, and alcohol use.

HOMA-IR, homeostasis model assessment of insulin resistance.

**Figure 2:**
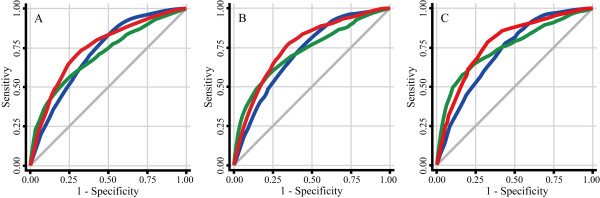
**ROC curves for different waist circumferences in relation to insulin resistance**. panel A: HOMA-IR ≥ 1.50; panel B: HOMA-IR ≥ 2.00; and panel C: HOMA-IR ≥ 2.50. ROC curves are shown for waist circumferences of 80 cm (blue line), 85 cm (red line), and 90 cm (green line). Logistic regression analysis was used with adjustment for age, rank in the Self-Defense Forces, smoking, and alcohol use. ROC, receiver operating characteristics; HOMA-IR, homeostasis model assessment of insulin resistance.

In the ROC curve analysis (Table 3), the highest value of Youden index was obtained for a cutoff point of 85 cm in waist circumference across different values of HOMA-IR. The multiple logistic regression analysis also indicated that the AUC was consistently the largest for a waist circumference of 85 cm (Figure 2).

**Table 3:** Optimal cutoffs of waist circumference in different HOMA-IR values from ROC curve analysis

HOMA-IR	Prevalence (%)	Cutoff of WC (cm)	Sensitivity (%)	Specificity (%)	Youden index
1.50	34.9	80	91.5	39.5	0.310
		85	70.4	70.0	0.403
		90	38.8	89.5	0.284
2.00	20.3	80	94.0	34.4	0.285
		85	78.0	64.5	0.425
		90	47.6	86.6	0.342
2.50	12.0	80	96.0	32.0	0.280
		85	83.8	61.3	0.451
		90	56.1	84.5	0.406
3.00	7.8	80	96.3	30.7	0.270
		85	85.3	59.3	0.446
		90	60.1	83.0	0.430

ROC, receiver operating characteristics; HOMA-IR, homeostasis model assessment of insulin resistance; WC, waist circumference.

## Discussion

The present study demonstrated a strong, linear relationship between waist circumference and insulin resistance as expressed by HOMA-IR in a large population of Japanese men. Both geometric means and prevalence odds of elevated HOMA-IR were progressively greater in proportion to the size of waist circumference. The increase was evident even in men with the average size of waist, i.e., 80–84 cm. It was also found that 85 cm of waist circumference was an optimal cutoff for predicting insulin resistance. The finding adds to evidence for optimality of the cutoff for waist circumference proposed for Japanese men.

Obesity has been known to be positively related to insulin resistance. Increased secretion of free fatty acids, inflammatory cytokines and decreased secretion of adiponectin are molecules mediating obesity and insulin resistance [18,19]. Few studies have directly addressed the relationship between waist circumference and insulin resistance or hyperinsulinemia [20,21]. A small cross-sectional study reported a linear increase in the prevalence of hyperinsulinemia across the deciles of waist circumference in 185 healthy men in Canada [20]. In a cross-sectional study of 2746 volunteers aged 18–72 years, including 798 men, waist circumference was strongly correlated with HOMA-IR [21]. The present study was the ever largest study of men examining the relation between waist circumference and HOMA-IR. It is also notable that even waist circumference of 80–84 cm was associated with an evident increase in the prevalence odds of insulin resistance. The finding is in agreement with the notion that obesity-related risk is present at much lower levels of obesity in Asians as compared with Caucasians [22].

Insulin resistance is an obesity-related condition preceding the development of impaired glucose tolerance and type 2 diabetes. Insulin resistance, through suppression of glucose uptake in skeletal muscle and increase in hepatic glucose production, causes hyperglycemia [1]. Insulin resistance expressed by HOMA-IR is well in agreement with that evaluated directly by the euglycemic clamp method [15,16], and is generally accepted as a valid method in epidemiological surveys [23]. However, there is no clear cutoff for the definition of insulin resistance based on HOMA-IR. Insulin resistance based on HOMA-IR has been defined differently in different studies. For instance, insulin resistance was defined as HOMA-IR of ≥ 3.80, which corresponded to the 90th percentile in healthy subjects, in Spain [24], and a value of 4.00 or greater was used in a Swedish study [21]. HOMA-IR ≥ 1.73 was used in a Japanese study [25]. We defined insulin resistance arbitrarily as HOMA-IR greater than the 80th percentile (≥ 2.00) in examining the relationship with waist circumference. Repeated analyses using HOMA-IR cutoffs of 1.50 and 2.50 showed almost the same results in terms of the prevalence odds (data not shown). Different values of HOMA-IR were used in the ROC curve analysis of searching for an optimal cutoff for waist circumference. It should be noted that the optimal cutoff point was consistently 85 cm for any values of HOMA-IR. However, the discordance between abdominal obesity (≥ 85 cm) and insulin resistance accounted for no less than 33%. This value was almost equal to or slightly greater than those reported for the discordance between metabolic syndrome and insulin resistance among Americans and Turks [26]. Thus, although waist circumference was found to be strongly related to insulin resistance, it is unlikely that Japanese men have less confounders in the relationship under study as compared with other ethnicities.

More emphasis has been placed on waist circumference as an estimate of visceral adiposity rather than body mass index in the prevention of obesity-related diseases. In the present study population, waist circumference and body mass index were highly correlated with each other (Pearson correlation coefficient 0.86), and body mass index was as strongly associated with HOMA-IR as waist circumference. For example, an increase of one SD in body mass index was associated with an 42.0% increase in HOMA-IR while the corresponding value for waist circumference was 43.7%.

A strength of the present study was that the study population was very large and relatively homogeneous in terms of the social background. There were several weaknesses to be discussed. The present study was based on estimated measures for both visceral adiposity and insulin resistance, and these estimates necessarily suffered some inaccuracy which may have attenuated the association between the two. Interpretation of causality is difficult in cross-sectional studies. It is possible that insulin resistance or related conditions may increase visceral adiposity. Furthermore, inflammation and atherosclerosis are linked to insulin resistance, and these conditions may confound the relationship under study. Our study subjects were not representative of middle-aged Japanese men. Age was limited to a small range, and the subjects were those who had remained in the SDF until the age of 50 years on average. It is possible that the study subjects differed from the general population with respect to both waist circumference and insulin resistance, although body mass index in the study population did not differ from that of the general population. In the National Nutrition Survey in 2000, means of body mass index for men aged 40s and 50s were 23.5 and 23.6, respectively [27]. It is also a limitation that the study did not include women. Women, particularly middle-aged ones, are very few in the SDF. Finally, it may be argued that inclusion of men with oral medication for diabetes may have distorted the association between waist circumference and HOMA-IR. However, such men still had higher values of HOMA-IR as compared with the others (geometric means 2.02 versus 1.17).

## Conclusion

A large cross-sectional study of Japanese men showed a strong, linear relationship between waist circumference and insulin resistance. The ROC curve analysis consistently indicated that an optimal cutoff of waist circumference was 85 cm in association with insulin resistance. The findings lend a strong support for the Japanese criterion for abdominal obesity in men in the metabolic syndrome.

## Abbreviations

AUC: area under curve; HOMA-IR: homeostasis model assessment of insulin resistance; IDF: International Diabetes Federation; IQR: interquartile range; ROC: Receiver operating characteristics; SDF: Self-Defense Forces; WC: waist circumference.

## Competing interests

All authors declare that the answer to the questions on your competing interest form are all No and therefore have nothing to declare.

## Authors' contributions

ST and SK designed the study, supervised the collection of the data, analyzed the data, interpreted the results, and prepared the first draft of the report. SY, TH, and HA participated in the collection of data and interpreted the results. KO designed the study and interpreted the results. All authors participated in the critical revision of the report and approved the final report.

## Pre-publication history

The pre-publication history for this paper can be accessed here:


